# Food Additives as Novel Influenza Vaccine Adjuvants

**DOI:** 10.3390/vaccines7040127

**Published:** 2019-09-24

**Authors:** Huapeng Feng, Makoto Yamashita, Li Wu, Tiago Jose da Silva Lopes, Tokiko Watanabe, Yoshihiro Kawaoka

**Affiliations:** 1 Division of Virology, Department of Microbiology and Immunology, Institute of Medical Science, University of Tokyo, Tokyo 108-8639, Japan; hpfeng@ims.u-tokyo.ac.jp (H.F.); yamakoto@ims.u-tokyo.ac.jp (M.Y.); lwu19870426@gmail.com (L.W.); 2 Department of Pathobiological Sciences, School of Veterinary Medicine, University of Wisconsin-Madison, Madison, WI 53711, USA; tiagojose.dasilvalopes@wisc.edu; 3 Department of Special Pathogens, International Research Center for Infectious Diseases, Institute of Medical Science, University of Tokyo, Tokyo 108-8639, Japan

**Keywords:** food additives, influenza, vaccine, adjuvants, mice

## Abstract

Influenza is a major threat to public health. Vaccination is an effective strategy to control influenza; however, the current inactivated influenza vaccine has mild immunogenicity and exhibits suboptimal efficacy in clinical use. Vaccine efficacy can be improved by the addition of adjuvants, but few adjuvants have been approved for human use. To explore novel and effective adjuvants for influenza vaccines, here we screened 145 compounds from food additives approved in Japan. Of these 145 candidates, we identified 41 compounds that enhanced the efficacy of the split influenza hemagglutinin (HA) vaccine against lethal virus challenge in a mouse model. These 41 compounds included 18 novel adjuvant candidates and 15 compounds with previously reported adjuvant effects for other antigens but not for the influenza vaccine. Our results are of value to the development of novel and effective adjuvanted influenza or other vaccines for human use.

## 1. Introduction

Influenza viruses are categorized into four types: type A, type B, type C, and type D. Influenza A and B viruses (H1N1, H3N2, B/Yamagata, or B/Victoria lineage) are the main pathogens that cause seasonal influenza epidemics every year. Seasonal influenza is responsible for up to 650,000 deaths annually according to World Health Organization (WHO) estimates [1]. Vaccination is an effective strategy to combat influenza virus infection in humans and animals [2,3,4,5]. The inactivated influenza vaccine is the most widely used annual vaccine throughout the world [6,7]; however, the efficacy of inactivated influenza vaccines is modest in adults and worse in the elderly [8,9].

An adjuvant is added to, or given in conjunction with, a vaccine antigen to enhance the specific immune response. For the influenza vaccine, adjuvants are added to improve their immunogenicity, to enhance efficacy in the elderly, and to reduce the amount of virus antigens required for protection or the number of immunizations needed. In addition, adjuvants can extend the protection period and broaden reactivity to antigenically different strains, which would be beneficial when there is an antigenic mismatch between the influenza vaccine strains and the circulating viruses [10]. Aluminum salt, which is referred to as ‘alum’ and is the most widely used adjuvant for human vaccines, and squalene-based oil-in-water emulsions (e.g., MF59 and AS03) have been licensed as adjuvants for human influenza vaccines [11,12]; however, the efficacy of adjuvanted influenza vaccines remains suboptimal [13]. Additionally, concerns about the safety of adjuvants have been raised, such as recent reports regarding an increased incidence of narcolepsy in children when the squalene-based adjuvant AS03 was used in a pandemic influenza vaccine [14,15]. Therefore, the development of safe, non-reactogenic adjuvants that elicit strong protective immunity for the influenza vaccine is urgently needed.

Food additives are substances added to food to assist in its processing or to improve certain characteristics, such as preserving flavor or enhancing taste and appearance. The food additive saponin has been used as an adjuvant for veterinary vaccines and experimental vaccines for many years [16], and saponin-based adjuvants, including QS21, ISCOMATRIX, Matrix M, and GPI-0100 are entering clinical trials [17,18,19,20]. Therefore, screening food additives for adjuvanticity is a valid approach to identifying novel adjuvants. To explore adjuvant candidates that exhibit strong protective immunity against influenza virus infection, here we screened 145 food additives approved in Japan for human use for adjuvant effects with the current seasonal influenza vaccine in mice. We identified 41 compounds that enhanced influenza virus-specific antibody responses and the efficacy of commercial influenza HA vaccines against the lethal challenge of the influenza virus. Our findings will facilitate the development of novel and effective adjuvanted human influenza vaccines and other vaccines. In addition, food additives approved for humans could be used as animal vaccine adjuvants to improve vaccine efficacy and food safety.

## 2. Materials and Methods

### 2.1. Cells and Viruses

Madin-Darby canine kidney (MDCK) cells were maintained in minimum essential medium (MEM) (Gibco) supplemented with 5% newborn calf serum (Sigma) at 37 °C in 5% CO_2_. MDCK cells were used for plaque assays to titrate viruses.

Mouse-adapted A/California/04/2009 virus (H1N1; MA-CA04), generated in our laboratory as previously described [21], was used to challenge mice. A/California/07/2009 virus (H1N1; CA07), which was isolated early in the 2009 pandemic and is one of the components of the split influenza HA vaccine, was used as an antigen for the ELISA to determine the virus-specific antibody titers of sera obtained from the immunized mice.

### 2.2. Influenza Vaccines and Compounds Used For The Screen

Trivalent and quadrivalent split influenza HA vaccines were obtained from DENKA SEIKEN Co., Ltd. (Tokyo, Japan). The trivalent influenza HA vaccine (for the 2014–2015 season), which contains the HA proteins (equivalent to 30 μg of HA protein for each virus included in a vaccine vial) of CA07 (H1N1), A/New York/39/2012 (H3N2), and B/Massachusetts/2/2012 (Yamagata lineage), was used for the primary screen to examine the viral-specific antibody titers in sera obtained from the immunized mice. The quadrivalent split influenza HA vaccine (for the 2015–2016 season), which contains the HA proteins (equivalent to 30 μg of HA protein for each virus included in a vaccine vial) of CA07 (H1N1), A/Switzerland/9715293/2013(H3N2), B/Phuket/3073/2013 (Yamagata lineage), and B/Texas/2/2013 (Victoria lineage), was used for the secondary screen to examine the protective efficacy of the immunized mice. The quadrivalent split influenza HA vaccine (for the 2016–2017 season), which contains the HA proteins (equivalent to 30 μg of HA protein for each virus included in a vaccine vial) of CA07 (H1N1), A/Hongkong/4801/2014 (H3N2), B/Phuket/3073/2013 (Yamagata lineage), and B/Texas/2/2013 (Victoria lineage), was used for part of the secondary screen and for testing virus replication in the immunized mice after virus challenge. For compound testing, aluminum hydroxide gel Alhydrogel^®^ adjuvant 2% (alum), purchased from InvivoGen, was used as a positive control (antigen: alum (*v*/*v*) = 1:1) (approximately equal to 500 μg of alum/dose). All other food additive compounds tested (selected after excluding enzymes, salts, elemental substances, proteins, biological small compounds, wax, carbon hydrides, and soil) were purchased from the companies listed in Table S1. These compounds were suspended in phosphate-buffered saline (PBS; without calcium or magnesium) at concentrations of 10 mg/mL or 10 µL/mL and were sonicated in a water bath for 15 min at room temperature to generate compound stocks. The compound stocks were then stored at −20 °C until use except for alum, which was stored at room temperature. Before being mixed with the split influenza HA vaccine, the suspensions or solutions were thawed and sonicated again for 5 min.

### 2.3. Immunization and Protection

Five-week-old female BALB/c mice were purchased from Japan SLC Inc.(Shizuoka, Japan) After one week of adaptation, the mice were immunized with a suboptimal dose of the influenza HA vaccine (0.01 µg/dose (2014–2015 season), 0.003 µg/dose (2015–2016 season), or 0.001 µg/dose (2016–2017 season) calculated on the basis of the amount of HA from CA07 (H1N1)) with or without compounds via intramuscular injection into the gastrocnemius muscle. Two weeks later, the mice were boost-immunized intramuscularly. On day 14 after the boost-immunization, blood was collected via the facial vein using a goldenrod animal lancet (5 mm), and sera were isolated for measuring virus-specific antibody titers. Since it was not possible to screen 145 compounds in mice simultaneously, the 145 compounds were divided into 15 sets (1–11 compounds per set) and alum was used as the positive control in each set.

In the secondary screen, the 59 compounds were divided into 10 sets (2–8 compounds per set) and alum was used as the positive control in each set; three weeks after the boost-immunization, the immunized mice were challenged intranasally, under anesthesia, with 10 equivalents of the dose required to kill 50% of infected mice (MLD_50_; equivalent to 6.8 × 10^5^ PFU/50 µL/mouse for this work) of MA-CA04 virus. Body weight and survival were monitored daily for 14 days after virus challenge. Mice that lost more than 25% of their original body weight were euthanized. Three mice per group were used for the primary screen except for the sage oil group (*n* = 4) and four mice per group were used in the secondary screen. To determine virus titers in mice, organs were harvested on days 3 and 6 post-challenge from three mice per group, homogenized, and titrated on MDCK cells by using a plaque assay as described previously [22]. The number of biological replicates was three and four for the primary and the secondary screens, respectively, except for the sage oil group, for which the number was four in the primary screen. The number of biological replicates was three for the virus replication experiment. All experiments were conducted once; however, since the antibody titers for the 59 hit compounds identified in the primary screen were also measured in the secondary screen, the number of repeats for the antibody titer measurement of the 59 compounds was two.

### 2.4. Measurement of Virus-Specific Antibody Titers

Virus-specific antibody titers in sera were determined using a modified ELISA as previously described [23,24]. Briefly, 96-well ELISA plates (IWAKI) were coated with 6 µg/mL of inactivated and purified CA07 virus solution overnight at 4 °C (50 µL/well). The plates were then blocked with 200 µL of 20% blocking one (Nacalai) in water at room temperature for 1 h. After blocking, the plates were washed once with PBS containing 0.05% Tween-20 (PBS-T), and then 2-fold serially diluted serum samples were added to the plates, followed by a 1 h incubation at room temperature. Bound IgG was detected by using peroxidase-labeled goat anti-mouse IgG (gamma) antibody, F (ab’) 2 fragment (Kirkegaard and PerryLaboratory Inc.; Gaithersburg, MD, USA). After the plates were washed four times with PBS-T, 100 µL of 2,2′-azino-bis (3-ethylbenzothiazoline-6-sulphonic acid) diammonium salt substrate solution was added to each well to initiate the color reaction, and the optical density (OD) was measured at a wavelength of 405 nm. The antibody titer was defined as the reciprocal of the highest serum dilution that produced an OD_405_ > 0.1 after correcting for the negative serum control [25].

### 2.5. Statistics

We used R [26] and lme4 [27] to perform a linear mixed effects analysis of the body weight data, which were normalized to the initial weight of each individual animal. As fixed effects, we used the different treatment groups (i.e., vaccine alone, vaccine plus compound, and vaccine plus alum), and the time of measurement (with an interaction term between those fixed effects). As random effects, we had intercepts for the individual animals. We used the lsmeans [28] package to compare the groups at different timepoints for each model separately, and the *p* values were adjusted using Holm’s method. For the comparisons of virus titers, we used one-way ANOVA, followed by Dunnett’s tests, with *p* values adjusted using Holm’s method. Each timepoint was analyzed separately. For the analysis of the survival data, we used a Log-rank test, comparing the vaccine plus compound or alum groups to the vaccine alone group. We used OASIS 2 [29] software for this analysis. *p* values < 0.05 were considered statistically significant.

### 2.6. Ethics

All experiments with mice were performed in the biosafety level 2 containment laboratory in the Institute of Medical Science, the University of Tokyo (Tokyo, Japan) in accordance with the Regulations for Animal Care of the University of Tokyo and the Guidelines for Proper Conduct of Animal Experiments by the Science Council of Japan, and were approved by the Animal Experiment Committee of the Institute of Medical Science, the University of Tokyo (approval no. PA14-38).

## 3. Results

### 3.1. Identification of 59 Compounds that Enhance the Humoral Responses to an Influenza Vaccine in Mice

To explore novel adjuvants for commercially available split influenza HA vaccines, we conducted a chemical screen in a mouse model by using 145 compounds selected from the approved food additives in Japan to identify compounds that could enhance influenza virus-specific antibody responses. Commercially available alum adjuvant was used as a positive control, as described in Materials and Methods, because alum is the most frequently used adjuvant worldwide and has been used in many clinical studies [12]. First, we performed an optimization experiment to determine the optimal dose of the HA vaccine (for the 2014–2015 season; the trivalent influenza HA vaccine, which contains the HA proteins of CA07 (H1N1), A/New York/39/2012 (H3N2), and B/Massachusetts/2/2012 (Yamagata lineage)) for our screen. We found that a dose of 0.01 µg of the HA vaccine (0.01 μg of HA for each virus/dose) elicited virus-specific antibodies only when the alum adjuvant was administered to mice together with the vaccine, whereas a dose of 0.03 µg of the HA vaccine induced virus-specific antibodies both in the presence and absence of alum (Figure S1), indicating that a dose of 0.01 µg of the HA vaccine was appropriate to use to immunize mice for our purposes.

**Figure 1:**
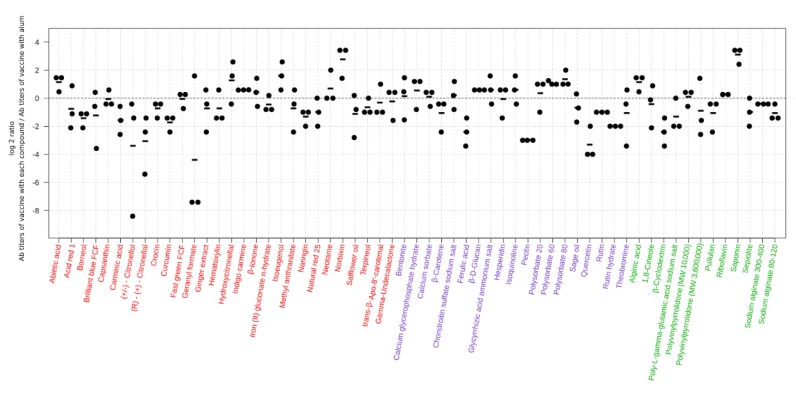
Virus-specific antibody titers induced in mice by the 59 hit compounds in combination with the HA vaccine. Six-week-old BALB/c mice were immunized with the influenza HA vaccine with or without compounds twice with a two-week interval between the vaccinations. Blood samples were collected two weeks after the second immunization. Virus-specific antibodies were measured by using an ELISA with inactivated and purified CA07 virus as the coating antigen. Depicted are the antibody titers obtained from the mice immunized with the vaccine with the hit compounds. Each dot represents one mouse, and the individual antibody titers were divided by the average of the antibody titers of the mice immunized with the vaccine plus alum in the same batch. This procedure normalizes the values of the animals immunized with candidate compounds to their respective positive controls, and the log transformation aids in the representation of the values. Values above zero indicate that the antibody titers of the mice treated with the vaccine plus the hit compounds were higher than those of their positive controls. The black horizontal line represents the mean antibody titers from individual mice (*n* = 3, except sage oil *n* = 4). The dotted line represents the reference vaccine plus alum. The status of the 59 hits is indicated by the color used for the compound names: red compound names represent novel adjuvant candidates; blue compound names represent novel adjuvant candidates for the influenza vaccine; compounds with green names indicate that their adjuvant effect for the influenza vaccine has been reported previously. MW: molecular weight.

For the primary screen, mice were immunized twice with PBS, compound alone (100 µg/dose), HA vaccine alone (0.01 μg of HA for each virus/dose), or HA vaccine plus compound (100 µg/dose) via intramuscular administration in a 100 μL volume with a two-week interval between the vaccinations. For saponin, one of the 145 compounds tested in the primary screen, a lower dose (10 µg/dose) was used for the immunizations because intramuscular administration of 100 µg/dose of saponin resulted in severe side effects in the mice: that is, all of the mice died by day 3 post-injection. Two weeks after the boost immunization, serum samples were collected from the immunized mice. We measured the titers of virus-specific antibody against CA07 virus (which is one of the components of the HA vaccine we tested) using an ELISA, and found that no antibodies against the CA07 virus were detected in the PBS or compound alone groups. Mice immunized with the HA vaccine alone produced no or low levels of virus-specific antibodies; the antibody titers ranged from <10 to 320 (Table 1 and Table S1). Therefore, we defined hits as compounds that induced a mean antibody titer of >320 in combination with the HA vaccine (for the 2014–2015 season) in the primary screen; 59 compounds that enhanced antibody production compared with the HA vaccine alone were selected on the basis of these criteria (Table 1 and Table S1). These 59 hit compounds included 28 novel adjuvant candidates, 19 novel adjuvant candidates for the influenza vaccine (i.e., their adjuvanticity had been reported for other antigens but not for the influenza vaccine), and 12 previously reported adjuvant candidates for the influenza vaccine (Figure 1, Table 1, and Table S1). Among these hits, norbixin elicited the highest antibody titer in combination with the HA vaccine. Twenty-nine hits induced similar or higher antibody titers compared with that elicited by alum, although there was no statistically significant difference except for saponin.

The 59 hits comprised several categories of food additive: 7 antioxidants (e.g., β-d-glucan, hesperidin, rutin, and rutin hydrate), 19 colors (e.g., β-carotene, norbixin, and riboflavin), 13 flavors (e.g., hydroxycitronellal, theobromine, and γ-undecalactone), 7 emulsifiers (e.g., chondroitin sulfate sodium salt, β-cyclodextrin, and polysorbate 20), 2 sweeteners (glycyrrhizic acid ammonium salt and neotame), 2 preservatives (abietic acid and calcium sorbate), 4 thickeners (calcium glycerophosphate hydrate, pullulan, and sodium alginate 80–120), 6 stabilizers (calcium glycerophosphate hydrate, chondroitin sulfate sodium salt, and polyvinylpyrrolidone (MW 3,600,000)), 2 acidity regulators (abietic acid and sepiolite), 2 anticaking agents (bentonite and sepiolite), and one foaming agent (saponin) (Table 1 and Table S1). There was some overlap because some food additives have more than one function. Most hits were from the color additive category. These results indicate that many kinds of food additives can act as vaccine adjuvants.

### 3.2. Identification of 41 Compounds that Enhance the Protective Efficacy of Influenza Vaccine against Lethal Virus Challenge in Mice

For the 59 compounds selected in the primary screen, we next conducted a secondary screen to examine whether these compounds enhance the protective efficacy of the HA vaccine against lethal challenge in mice. The HA vaccine was updated to the quadrivalent split influenza HA vaccine (for the 2015–2016 or 2016–2017 influenza season) for the secondary screen and the appropriate doses were determined to be 0.003 μg/dose for the 2015–2016 season vaccine (0.003 μg of HA for each virus/dose) and 0.001 μg/dose for the 2016–2017 season vaccine (0.001 μg of HA for each virus/dose). Two weeks after the boost immunization, serum samples were collected from the immunized mice and the virus-specific antibody titers were measured (Table 1). All 59 compounds plus the HA vaccine exhibited higher antibody titers compared with the vaccine alone group in the secondary screen; however, some of compounds (i.e., bentonite, (+/−)-citronellol, (R)-(+)-citronellal, β-ionone poly-L-γ-glutamic acid sodium salt, and sage oil) induced lower antibody titers compared with those in the primary screen (Table 1).

The immunized mice were challenged with 10 MLD_50_ of the MA-CA04 virus one week after blood collection (three weeks after the boost immunization), and then body weight and survival rate were monitored daily for 14 days. Forty-one compounds showed protective efficacy similar or superior to that of alum on the basis of survival rates (Table 2). These 41 hit compounds comprised 18 totally novel adjuvant candidates, 15 novel adjuvant candidates for the influenza vaccine with previously reported adjuvant effects for other antigens but not for the influenza vaccine, and 8 previously reported adjuvant candidates for the influenza vaccine (Table 1 and Table 2), namely β-cyclodextrin [30,31], poly-L-γ-glutamic acid sodium salt [32,33], polyvinylpyrrolidone (MW 3,600,000) [34], pullulan [35], riboflavin [36], saponin [37,38,39], sepiolite [40], and sodium alginate 80–120 [41] (Table 1, Figure 1, and Table S1).

**Figure 2:**
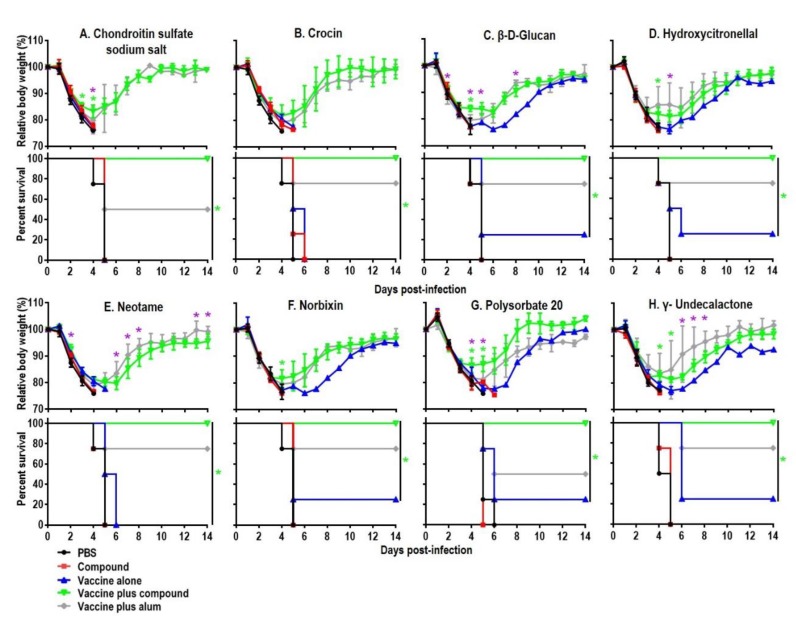
Body weight changes and survival rates of immunized mice after lethal challenge. Six-week-old BALB/c mice were mock-immunized with phosphate-buffered saline (PBS) or compounds only, or they were immunized with the HA vaccine alone or the compound-adjuvanted HA vaccine twice with a two-week interval. Mice were intranasally challenged with 10 MLD_50_ of MA-CA04 virus three weeks after the second immunization. Body weight and survival were monitored for 14 days. The body weight data shown are means and standard deviations (*n* = 4). Green asterisks indicate a significant difference between the vaccine alone and the vaccine plus compound groups (vaccine plus compound versus vaccine alone); purple asterisks indicate a significant difference between the vaccine plus alum and the vaccine plus compound groups (vaccine plus alum versus vaccine plus compound); * *p* < 0.05.

Among the 18 novel hits, 8 compounds (i.e., carminic acid, crocin, hydroxycitronellal, methyl anthranilate, neotame, norbixin, terpineol, and γ-undecalactone) completely protected the mice when immunized with HA vaccine from lethal challenge with MA-CA04 virus, whereas all mice given PBS or the compound alone showed body weight loss after virus challenge and died by 7 days post-challenge (Figure 2, Table 2, Figure S2, and Table S2). Among these compounds, neotame, norbixin, and γ-undecalactone induced similar or higher titers of virus-specific antibody relative to those induced by alum in the secondary screen (Table S2). In addition, five other compounds (i.e., carminic acid, crocin, hydroxycitronellal, methyl anthranilate, and terpineol) induced lower antibody titers in the immunized mice relative to alum but provided protective efficacy that was comparable to or better than that of alum (Table 1 and Figure S2). Notably, all seven emulsifiers from the primary screen enhanced the protective efficacy of influenza vaccine in the secondary screen (Table 1 and Table 2).

### 3.3. Effect of Immunizing Mice with the HA Vaccine Together with the Top Five Compounds on the Replication of the Challenge Virus in the Respiratory Tract

To examine the effects of promising adjuvant candidates on virus replication in the immunized mice after challenge, we selected 5 of the 41 positive compounds (i.e., β-d-glucan, neotame, norbixin, polysorbate 20, and γ-undecalactone) that were novel adjuvant candidates for the influenza vaccine, induced higher antibody titers than alum, and showed complete protective efficacy and less weight loss (Figure 2 and Table 2), for further testing. Six mice per group immunized with the HA vaccine and the respective compound were challenged three weeks after the boost immunization with 10 MLD_50_ of MA-CA04 virus, and organ samples (i.e., nasal turbinates (NT) and lungs) were collected from the sacrificed mice on days 3 and 6 post-infection for virus titration. On day 3 post-challenge, high titers of viruses were recovered from both the nasal turbinates and the lungs of all mice (Table 3). In contrast, on day 6 post-challenge, no or low titers of virus were detected from the nasal turbinates and lungs of some of the adjuvant groups (i.e., ‘vaccine plus neotame’ and ‘vaccine plus polysorbate 20′) compared with the vaccine alone group, although there was no significant difference (Table 3). These results suggest that some of the candidate adjuvants, such as neotame and polysorbate 20, might facilitate the rapid clearance of viruses from mice even though they did not protect mice from replication of the challenge virus.

## 4. Discussion

Food additives have been widely used by humans for a long time. In this study, we screened 145 food additives approved in Japan for novel compounds with adjuvanticity in a mouse model and obtained 41 hits that were comparable to alum on the basis of survival rates, including 18 totally novel adjuvant candidates, 15 novel adjuvant candidates for influenza vaccine, and 8 previously reported adjuvants for influenza vaccine [30,31,32,33,34,35,36,37,38,39,40,41]. Of the 41 hits, the top five compounds (i.e., β-d-glucan, neotame, norbixin, polysorbate 20, and γ-undecalactone) were examined for their effects on virus replication in immunized mice after virus challenge, and none of them protected mice from virus replication. This might be because the amount of HA vaccine was too low to inhibit virus replication in mice since we used the minimum amount of the HA vaccine in this study to see the adjuvant effects of the compounds; however, the immunization of mice with such a low dose of HA vaccine together with the hit compounds might be sufficient to facilitate the rapid clearance of the virus from the mice, resulting in 100% protection.

Recent studies suggest that adjuvants act to elicit immune responses by utilizing one or more of the following mechanisms: the depot effect, induction of cytokine responses and gene expression profile changes, immune cell recruitment, enhancement of antigen uptake and dendritic cell maturation, host dsDNA release, innate signaling pathway and inflammasome activation, and Th1 and Th2 immune responses and T follicular helper-cell generation [10,31]. Although the mechanism of action for most of the hit compounds identified in this study remains unknown, our hit compounds could be used to develop combination adjuvants, which is one of the most promising approaches to improving adjuvant efficacy and safety. GlaxoSmithKline Biologicals has developed several Adjuvant Systems, such as AS01, AS02, AS03, and AS04, in which known adjuvants are combined to induce innate and/or adaptive immune responses [42,43,44]. Further studies to identify the mechanisms of action of the adjuvant candidates identified in this screen would allow us to develop novel adjuvant systems by combining several adjuvant candidates with different mechanisms of action.

The hit compounds identified in this study are not only useful for the development of human vaccines but also for improving animal vaccines. Veterinary vaccines are important for animal and public health. Vaccination of animals can reduce animal suffering, ensure efficient production of food animals for human consumption, and decrease the amount of antibiotic use, thereby contributing to food safety [45]. There are several advantages to using food additives as animal vaccine adjuvants, the most important of which is to improve food safety. Veterinary vaccines use large quantities of adjuvants that are not yet approved for use in human vaccines. Adjuvant-induced injection site reactions and residues are problematic for vaccines administered to food-producing animals (e.g., vaccines for cattle, sheep, swine, chickens, and ducks) because they decrease the meat quality for human consumption or trade [46]. Therefore, using food additives approved for human use as food animal vaccine adjuvants instead of adjuvants with unknown safety profiles should improve vaccine efficacy and food safety. Moreover, the use of food additives as animal adjuvants may facilitate approval of such adjuvanted vaccines for animal clinical use given their established safety profiles in humans [47].

There was a large variation in the virus-specific antibody titers for the alum control across the screens (Table 1, Tables S1 and S2, and Figure S3). In this study, the 145 compounds were divided into 15 sets in the primary screen and alum was used as a positive control in each set. The antibody titers for the vaccine plus alum group showed variation presumably due to differences in the batches of mice used in each set and experimental variations. Additionally, to see the adjuvant effect of the compounds, we used a low amount of the HA vaccine, which could also have led to variations in the antibody responses. Given the performance of the alum, some compounds could have been false positives or false negatives in the primary screen. Indeed, some of the compounds (i.e., bentonite, (+/−)-citronellol, (R)-(+)-citronellal, β-ionone poly-L-γ-glutamic acid sodium salt, and sage oil) induced lower antibody titers in the secondary screen compared with those in the primary screen (Table 1), although this variation could have been due to the fact that we used HA vaccines from different influenza seasons (one from the 2015–2016 season and the other from the 2016–2017 season) and a lower amount per dose was used for the secondary screen (0.003 μg/dose or 0.001 μg/dose versus 0.01 μg/dose in the primary screen).

No adjuvant is suitable for all antigens; therefore, the selection of an appropriate adjuvant is an important step in the development of novel vaccines. The present study has identified a number of promising adjuvant candidates for influenza vaccine from food additives in mice. These adjuvant candidates could also be used for the development of novel universal influenza vaccines based on the purified HA stem or matrix 2 ectodomain (M2e) proteins since these protein antigens possess weak immunogenicity [48,49]. They may also be useful for the development of vaccines against other pathogens, food animal vaccines, or anticancer vaccines. We hope our efforts to identify novel adjuvants will help other researchers and vaccine companies to develop novel and effective adjuvanted vaccines.

## 5. Conclusions

Vaccine efficacy can be improved by the addition of adjuvants. In this study, we screened 145 compounds from food additives approved in Japan and identified 41 compounds that exhibited an adjuvant effect in mice when used with the split influenza HA vaccine. These compounds included 18 novel adjuvant candidates and 15 compounds with previously reported adjuvant effects for other antigens but not for the influenza vaccine. These findings are valuable to the development of novel and effective adjuvanted influenza or other vaccines.

## Figures and Tables

**Table 1:** Virus-specific antibody titers in sera from immunized mice for the 59 hit compounds identified in the primary screen ^a^.

Compound	Mean of Ab Titer-Primary Screen ^b^				Ab Titer Ratio-Primary Sceen ^d^ (Compounds/Alum)	Mean of Ab Titer-Secondary Screen ^b^				Ab Titer Ratio-Secondary Sceen ^d^ (Compounds/alum)	Function in Food	Status ^e^
	Compound	Vaccine Alone	Vaccine and Compound	Vaccine and Alum^c^		Compound	Vaccine Alone	Vaccine and Compound	Vaccine and Alum ^c^			
Abietic acid	<10	<10	533.33	233.33	2.29	<10	80, 160 ^f^	1280.00	560.00	2.29	Preservative, acidity regulator	a
Acid red 1	<10	10.00 ^f^	1173.33	1386.67	0.85	<10	205.00	1760.00	2240.00	0.79	Color	a
Alginic acid	<10	<10	533.33	233.33	2.29	<10	205.00	1280.00	2240.00	0.57	Thickener, stabilizer	c
Bentonite	<10	<10	346.67	233.33	1.49	<10	<10	140.00	600.00	0.23	Anticaking agent	b
Borneol	<10	10 ^f^	533.33	1386.67	0.38	<10	380.00	1920.00	2560.00	0.75	Flavor	a
Brilliant blue FCF	<10	<10	1333.33	1920.00	0.69	<10	<10	990.00	600.00	1.65	Color	a
Calcium glycerophosphate hydrate	<10	<10	1920.00	1120.00	1.71	<10	<10	4800.00	1440.00	3.33	Thickener, gelling agent, stabilizer	b
Calcium sorbate	<10	<10	2133.33	1920.00	1.11	<10	<10	3520.00	1440.00	2.44	Preservative	b
Capsanthin	<10	<10	1706.67	1706.67	1.00	<10	380.00	800.00	840.00	0.95	Color	a
Carminic acid	<10	<10	746.67	1920.00	0.39	<10	80, 40, 320 ^f^	960.00	1440.00	0.67	Color	a
β-Carotene	<10	<10	960.00	1706.67	0.56	<10	80, 160 ^f^	1120.00	560.00	2.00	Color	b
Chondroitin sulfate sodium salt	<10	<10	1493.33	1120.00	1.33	<10	<10	2880.00	1440.00	2.00	Emulsifier, stabilizer	b
1,8-Cineole	<10	10 ^f^	1386.67	1386.67	1.00	<10	<10	990.00	1440.00	0.69	Flavor	c
(+/−)-Citronellol	<10	<10	1280, 640 ^f^	1706.67	N/A	<10	<10	260.00	720.00	0.36	Flavor	a
(R)-(+)-Citronellal	<10	<10	333.33	1706.67	0.20	<10	<10	40, 80, 20 ^f^	1600.00	NA	Flavor	a
Crocin	<10	<10	1066.67	1706.67	0.63	<10	<10	480.00	720.00	0.67	Color	a
Curcumin	<10	<10	1066.67	3413.33	0.31	<10	380.00	480.00	840.00	0.57	Color	a
β-Cyclodextrin	<10	<10	373.33	1706.67	0.22	<10	<10	1440.00	1600.00	0.90	Emulsifier	c
Fast green FCF	<10	<10	2133.33	2133.33	1.00	<10	<10	440.00	600.00	0.73	Color	a
Ferulic acid	<10	<10	746.67	3413.33	0.22	<10	380.00	640.00	840.00	0.76	Antioxidant	b
Geranyl formate	<10	<10	2560 ^f^	853.33	N/A	<10	<10	1280, 1280, 1280 ^f^	1600.00	NA	Flavor	a
Ginger extract	<10	<10	2773.33	3413.33	0.81	<10	80, 40, 320 ^f^	1440.00	1440.00	1.00	Antioxidant	a
β-d-Glucan	<10	<10	2560.00	1706.67	1.50	<10	80, 160 ^f^	2560.00	560.00	4.57	Antioxidant	b
Glycyrrhizic acid ammonium salt	<10	<10	5973.33	3413.33	1.75	<10	80, 40, 320 ^f^	2560.00	1440.00	1.78	Sweetener	b
Hematoxylin	<10	<10	2560.00	3413.33	0.75	<10	380.00	1200.00	840.00	1.43	Color	a
Hesperidin	<10	<10	3840.00	3413.33	1.13	<10	80, 160 ^f^	2240.00	560.00	4.00	Antioxidant, nutrient supplement	b
Hydroxycitronellal	<10	<10	2773.33	853.33	3.25	<10	80, 40, 320 ^f^	1280.00	1440.00	0.89	Flavor	a
Indigo carmine	<10	<10	1280.00	853.33	1.50	<10	<10	485.00	600.00	0.81	Color	a
β-Ionone	<10	113.33	1493.33	960.00	1.56	<10	380.00	220.00	2560.00	0.09	Flavor	a
Iron (II) gluconate n-hydrate	<10	<10	853.33	1120.00	0.76	<10	<10	3040.00	1600.00	1.90	Color retention agent	a
Isoeugenol	<10	<10	2986.67	853.33	3.50	<10	<10	140.00	720.00	0.19	Flavor	a
Isoquinoline	<10	<10	1493.33	853.33	1.75	<10	80, 40, 320 ^f^	1200.00	1440.00	0.83	Color	b
Methyl anthranilate	<10	<10	693.33	853.33	0.81	<10	380.00	2240.00	2560.00	0.88	Flavor enhancer	a
Naringin	<10	320.00	2133.33	5120.00	0.42	<10	380.00	2240.00	840.00	2.67	Antioxidant	a
Natural red 25	<10	320.00	2986.67	5120.00	0.58	<10	300.00	440.00	2560.00	0.17	Color	a
Neotame	<10	40, 80 ^f^	2560.00	1280.00	2.00	<10	<10	720.00	720.00	1.00	Sweetener	a
Norbixin	<10	113.33	7680.00	960.00	8.00	<10	80, 40, 320 ^f^	2880.00	560.00	5.14	Color	a
Pectin	<10	320.00	640.00	5120.00	0.13	<10	<10	320.00	600.00	0.53	Vegetable gum, emulsifier	b
Poly-L-γ-glutamic acid sodium salt	<10	320.00	2560.00	5120.00	0.50	<10	<10	40, 40, 160 ^f^	1600.00	0.04	Flavor enhancer	c
Polysorbate 20	<10	40, 80 ^f^	1920.00	1280.00	1.50	<10	205.00	5760.00	2240.00	2.57	Emulsifier	b
Polysorbate 60	<10	40, 80 ^f^	2560.00	1280.00	2.00	<10	205.00	2560.00	2240.00	1.14	Emulsifier	b
Polyvinylpyrrolidone (MW 10,000)	<10	<10	533.33	480.00	1.11	<10	<10	652.50	1600.00	0.41	Emulsifier, stabilizer	c
Polysorbate 80	<10	40, 80 ^f^	3416.33	1280.00	2.67	<10	<10	4160.00	1600.00	2.60	Emulsifier	b
Polyvinylpyrrolidone (MW 3,600,000)	<10	<10	506.67	480.00	1.06	<10	<10	2720.00	1600.00	1.70	Emulsifier, stabilizer	c
Pullulan	<10	<10	960.00	1706.67	0.56	<10	380.00	720.00	840.00	0.86	Thickener, glazing agent	c
Quercetin	<10	320.00	640.00	5120.00	0.13	<10	<10	300.00	1600.00	0.19	Color	b
Riboflavin	<10	<10	2560.00	2133.33	1.20	<10	<10	2240.00	1440.00	1.56	Color	c
Rutin	<10	320.00	2560.00	5120.00	0.50	<10	<10	1320.00	1440.00	0.92	Color, antioxidant, nutrient supplement	b
Rutin hydrate	<10	320.00	1280.00	5120.00	0.25	<10	<10	720.00	720.00	1.00	Color, antioxidant, nutrient supplement	b
Safflower oil	<10	<10	693.33	1120.00	0.62	<10	80, 40, 320 ^f^	800.00	1440.00	0.56	Flavor	a
Sage oil ^g^	<10	320, 80 ^f^	746.67	960.00	0.78	<10	<10	160, 10 ^f^	1600.00	N/A	Flavor enhancer	b
Saponin	<10	<10	4266.67	480.00	8.89	<10	205.00	12160.00	2240.00	5.43	Foaming agent	c
Sepiolite	<10	320.00	2986.67	5120.00	0.58	<10	300.00	2080.00	2560.00	0.81	Acidity regulator, anticaking agent	c
Sodium alginate 300–400	<10	<10	1280.00	1706.67	0.75	<10	205.00	480.00	2240.00	0.21	Thickener, stabilizer	c
Sodium alginate 80–120	<10	<10	853.33	1706.67	0.50	<10	205.00	1280.00	2240.00	0.57	Thickener, stabilizer	c
Terpineol	<10	<10	853.33	1280.00	0.67	<10	380.00	2880.00	2560.00	1.13	Flavor	a
Theobromine	<10	<10	1333.33	1706.67	0.78	<10	80, 160 ^f^	1440.00	560.00	2.57	Flavor	b
trans-β-Apo-8′-carotenal	<10	<10	1280.00	1280.00	1.00	<10	380.00	1400.00	2560.00	0.55	Color	a
γ-Undecalactone	<10	113.33	960.00	960.00	1.00	<10	380.00	1920.00	840.00	2.29	Flavor	a

^a^ Six-week-old ca07 mice were intramuscularly immunized with the indicated immunogens (100 μL) twice with a two-week interval between immunizations. Three mice were used per group except for the sage oil group in the primary screen and four mice were used per group in the secondary screen. The serum samples were collected two weeks after the second immunization to measure the virus-specific antibody titers. ^b^ The virus-specific antibody titers (Ab titers) were determined by use of an ELISA with inactivated CA07 virus as the coating antigen. The OD was measured at a wavelength of 405 nm. The antibody titer was defined as the reciprocal of the highest serum dilution that produced an OD_405_ > 0.1 after correcting for the negative serum control. The values are the means of the three or four individual antibody titers per group. ^c^ For the primary screen, the 145 compounds were divided into 15 sets (1–11 compounds per set) and for the secondary screen, the 59 compounds were divided into 10 sets (2–8 compounds per set); alum served as the positive control in each set. ^d^ The Ab titer ratio = (mean of Ab titer (vaccine + compound))/(mean of Ab. titer (vaccine + alum)). N/A indicates not applicable because the mean antibody titer of the mice in the vaccine and compound group was <10. ^e^ a, novel adjuvants; b, novel adjuvants for the influenza vaccine, that is, their adjuvanticity has been reported for other antigens but not for influenza vaccine; c, previously reported hits for influenza vaccine. ^f^ Since the antibody titers in some animals were <10 among the four mice tested, only Ab titers ≥ 10 are shown. ^g^ Four mice were used in each group for sage oil in both the primary and secondary screens.

**Table 2:** Protective efficacy of the 41 hit compounds against lethal challenge of immunized mice ^a^.

Compound	Maximum Body Weight Loss % ± SD				Protective Efficacy (Survival/Total)				Enhanced Protective Efficacy (%) Compared to ^b^	
	Compound Alone	Vaccine Alone	Vaccine and Compound	Vaccine and Alum	Compound Alone	Vaccine Alone	Vaccine and Compound	Vaccine and Alum	Vaccine Alone	Vaccine and Alum
Abietic acid ^e,^*	23.6 ± 0.6	23.9	19.3 ± 2.8	20.4 ± 2.0	0/4	1/4	3/4	3/4	50	0
Brilliant blue FCF *	23.1 ± 2.0	24.3	18.2 ± 4.9	16.3 ± 3.7	0/4	0/4	3/4	3/4	75	0
Calcium glycerophosphate hydrate	23.6 ± 0.9	23.0 ± 1.5	23.1 ± 2.4	20.2 ± 5.1	0/4	0/4	3/4	2/4	75	25
Calcium sorbate	23.4 ± 0.4	23.0 ± 1.5	19.5 ± 3.1	20.2 ± 5.1	0/4	0/4	4/4	2/4	100	50
Carminic acid *	22.8 ± 1.9	22.7 ± 1.7	22.8 ± 1.4	15.4 ± 7.6	0/4	1/4	4/4	3/4	75	25
β-Carotene	24.7	23.9	17.4 ± 6.0	20.4 ± 3.7	0/4	1/4	4/4	3/4	75	25
Chondroitin sulfate sodium salt	22.6 ± 1.3	23.0 ± 1.5	16.8 ± 2.4	20.2 ± 5.1	0/4	0/4	4/4	2/4	100	50
(+/−)-Citronellol *	22.0 ± 1.8	22.4 ± 0.7	19.4 ± 2.1	20.9 ± 2.4	0/4	0/4	3/4	3/4	75	0
(R)-(+)-Citronellal *	24.1 ± 0.5	24.1	19.7 ± 4.3	17.6 ± 3.1	0/4	0/4	2/4	2/4	50	0
Crocin *	19.4 ± 0.7	21.6	18.2 ± 4.0	20.9 ± 2.4	0/4	0/4	4/4	3/4	100	25
β-Cyclodextrin	23.9	24.1	20.7 ± 2.8	17.6 ± 3.1	0/4	0/4	4/4	2/4	100	50
Fast green FCF *	22.1 ± 3.6	24.3	18.1 ± 3.2	16.3 ± 3.7	0/4	0/4	2/4	3/4	50	–25
Geranyl formate *	23.0	24.3	20.2 ± 5.0	16.3 ± 3.7	0/4	0/4	2/4	3/4	50	–25
β-d-Glucan	23.1 ± 0.6	23.9	17.4 ± 1.8	20.4 ± 2.0	0/4	1/4	4/4	3/4	75	25
Glycyrrhizic acid ammonium salt	24.7	23.5 ± 1.7	20.3 ± 3.4	15.4 ± 7.6	0/4	1/4	4/4	3/4	75	25
Hesperidin	21.8 ± 0.6	23.9	20.2 ± 2.2	20.4 ± 2.0	0/4	1/4	4/4	3/4	75	25
Hydroxycitronellal *	24.2 ± 0.7	23.5 ± 1.7	18.7 ± 2.2	16.1 ± 4.7	0/4	1/4	4/4	3/4	75	25
Indigo carmine *	24.2 ± 1.0	24.3	18.5 ± 3.6	16.3 ± 3.7	0/4	0/4	2/4	3/4	50	−25
Iron (II) gluconate n-hydrate *	23.6 ± 0.8	24.3	18.7 ± 2.1	18.6 ± 4.2	0/4	0/4	3/4	2/4	75	25
Isoeugenol *	22.3 ± 0.9	22.4 ± 0.7	21.7 ± 0.1	20.9 ± 2.4	0/4	0/4	2/4	3/4	50	−25
Isoquinoline	24.1	23.5 ± 1.7	21.2 ± 2.9	16.1 ± 4.7	0/4	1/4	3/4	3/4	50	0
Methyl anthranilate *	21.5 ± 3.2	22.1 ± 1.6	19.3 ± 1.0	20.0 ± 1.2	0/4	1/4	4/4	3/4	75	25
Naringin *	23.6 ± 0.8	22.9 ± 1.5	20.9 ± 2.9	16.4 ± 7.5	0/4	1/4	3/4	3/4	50	0
Neotame *	23.4 ± 0.8	22.4 ± 0.7	20.2 ± 2.4	20.9 ± 2.4	0/4	0/4	4/4	3/4	100	25
Norbixin *	23.9 ± 0.8	23.9	18.3 ± 3.3	20.4 ± 2.0	0/4	1/4	4/4	3/4	75	25
Pectin	24.5	24.3	21.8 ± 1.6	16.3 ± 3.7	0/4	0/4	2/4	3/4	50	−25
Poly-L-γ-glutamic acid sodium salt	23.7	24.1	18.9 ± 3.7	17.6 ± 3.1	0/4	0/4	2/4	2/4	50	0
Polysorbate 20	24.6	22.5	13.5 ± 2.8	18.8 ± 5.5	0/4	1/4	4/4	2/4	75	50
Polysorbate 60 ^c^	21.9 ± 0.6	22.5	19.5 ± 4.7	18.8 ± 5.5	0/4	1/4	2/4	2/4	25	0
Polysorbate 80	23.2	24.3	22.8 ± 1.5	20.0 ± 4.2	0/4	0/4	2/3^d^	2/4	66.7	16.7
Polyvinylpyrrolidone (MW 3,600,000)	22.6 ± 1.3	24.1	19.9 ± 3.0	17.6 ± 3.1	0/4	0/4	3/4	2/4	75	25
Pullulan	22.2 ± 0.8	22.9 ± 1.5	21.4 ± 1.7	16.4 ± 7.5	0/4	1/4	3/4	3/4	50	0
Riboflavin	23.2 ± 1.1	23.0 ± 1.5	22.0 ± 2.2	20.2 ± 5.1	0/4	0/4	4/4	2/4	100	50
Rutin	22.8 ± 1.4	23.0 ± 1.5	20.7 ± 2.5	20.2 ± 5.1	0/4	0/4	2/4	2/4	50	0
Rutin hydrate	21.7 ± 1.1	19.4 ± 0.7	18.0 ± 2.9	20.9 ± 2.4	0/4	0/4	4/4	3/4	100	25
Saponin	23.3	22.5	16.6 ± 3.9	18.8 ± 5.5	0/4	1/4	4/4	2/4	75	50
Sepiolite	22.2 ± 1.3	22.1 ± 1.6	19.0 ± 1.9	20.0 ± 1.2	0/4	1/4	3/4	3/4	50	0
Sodium alginate 80–120	24.2 ± 0.4	22.5	16.9 ± 2.3	18.8 ± 5.5	0/4	1/4	4/4	2/4	75	50
Terpineol *	22.7 ± 0.7	22.1 ± 1.6	19.2 ± 2.6	20.0 ± 1.2	0/4	1/4	4/4	3/4	75	25
Theobromine	23.1 ± 0.5	23.9	18.4 ± 2.4	20.4 ± 2.0	0/4	1/4	4/4	3/4	75	25
γ- Undecalactone *	23.8 ± 0.8	22.9 ± 1.5	18.8 ± 1.1	16.4 ± 7.5	0/4	1/4	4/4	3/4	75	25

^a^ Six-week-old BALB/c mice were intramuscularly immunized with the indicated immunogens (100 μL) twice at a two-week interval. Four mice were used per group. Three weeks after the second immunization, the mice were challenged with 10 MLD_50_ of the MA-CA04 virus. Body weight and survival were monitored daily for 14 days. ^b^ Enhanced protective efficacy here indicates the survival rate in the HA vaccine plus compound group compared with that in the HA vaccine alone group or the HA vaccine plus alum group. ^c^ Polysorbate 60 was identified as a hit in the secondary screen because it had a similar effect on weight loss and mortality as alum in the same batch. ^d^ One of four mice immunized with HA vaccine plus polysorbate 80 died after blood collection. ^e,^* Novel adjuvant candidates.

**Table 3:** Virus titers in the respiratory tract of immunized mice after challenge ^a^.

Immunogen	Mean Virus Titers (Log_10_ PFU/g) ± SD			
	Nasal Turbinates		Lungs	
	Day 3 p. i.	Day 6 p. i.	Day 3 p. i.	Day 6 p. i.
PBS	6.6 ± 0.2	5.9 ± 0.3	7.7 ± 0.1	6.7 ± 0.4
β-d-Glucan	6.4 ± 0.1	N/A ^b^, 5.3, 5.3	7.6 ± 0.1	N/A, 6.2, 5.5
Neotame	6.3 ± 0.1	6.2, 5.5, N/A	7.5 ± 0.2	7, 6.5, N/A
Norbixin	6.7 ± 0.2	5.6 ± 0.3	7.4 ± 0.3	6.4 ± 0.3
Polysorbate 20	6.8 ± 0.02	5.8 ± 0.3	7.5 ± 0.2	6.5 ± 0.4
γ-Undecalactone	6.7 ± 0.1	6.0 ± 0.3	7.4 ± 0.2	6.2 ± 0.2
Vaccine alone	6.3 ± 0.2	5.4 ± 0.7	7.4 ± 0.2	5.7 ± 1.0
Vaccine and β-d-glucan	6.0 ± 0.3	4.7 ± 0.9	7.2 ± 0.3	5.2 ± 0.7
Vaccine and neotame	6.1 ± 0.05	3.3 ± 0.4	7.5 ± 0.3	5.9, 4.4
Vaccine and norbixin	6.0 ± 0.3	4.2 ± 1.1	7.2 ± 0.3	4.4 ± 1.0
Vaccine and polysorbate 20	6.1 ± 0.1	5.4, 5.4	7.2 ± 0.2	5.7, 5.0
Vaccine and γ-undecalactone	6.2 ± 0.2	4.8 ± 0.3	7.0 ± 0.3	4.4 ± 1.2
Vaccine and Alum	6.3 ± 0.1	3. 6 ± 0.7	7.4 ± 0.3	2.8 ^c^

^a^ Six 6-week-old BALB/c mice per group were intramuscularly immunized with the indicated immunogens (100 μL) twice with a two-week interval between vaccinations and were then challenged with 10 MLD_50_ of MA-CA04 virus three weeks after the second immunization. Nasal turbinates and lungs were collected from mice on days 3 and 6 post-infection (p. i.) and virus titers were determined in MDCK cells by use of plaque assays. Individual titers were recorded when the virus was not detected in all three mice. ^b^ N/A indicates not applicable because the mice died on day 5 post-challenge. ^c^ The *p* value was <0.05 compared with the titer in the lungs of the mice immunized with vaccine alone after challenge. PFU: plaque-forming unit.

## Supplementary Materials

The following are available online at https://www.mdpi.com/2076-393X/7/4/127/s1. Figure S1: Determination of the appropriate antigen dose for the primary screen; Figure S2: Body weight changes and survival rates of immunized mice after lethal challenge (33 hit compounds); Table S1: The properties, antibody titers, and status of the food additives used in this study (Excel file); Table S2: The protective efficacy generated by immunization with each of 59 hit compounds and HA vaccine in mice (Excel file).
