# Supplementary material for: Food Additives as Novel Influenza Vaccine Adjuvants
Source: Vaccines (Basel). 2019 Sep 24;7(4):127. doi: 10.3390/vaccines7040127 (PMC6963695; doi:10.3390/vaccines7040127)
Supplement: Supplementary file 1 [file vaccines-07-00127-s001.zip › Supplementary Files/Feng_Vaccines_rev3_Sup Info.docx]

**1Supplementary Information**

Figure S1, Figure S2 and Figure S3

- Supplementary Figure legends
- Table S1. The properties, antibody titers, and status of the food additives used in this study (excel file)
- Table S2. The protective efficacy generated by immunization with each of 59 hit compounds and HA vaccine in mice (excel file)

**Supplementary Figure legends**

**Figure S1. Determination of the appropriate antigen dose for the primary screen.**

Six-week-old mice were immunized twice with a two-week interval between immunizations. Five doses (0.01 µg, 0.03 µg, 0.1 µg, 0.3 µg, and 1 µg) of HA vaccine (2014–2015 season) with or without alum were evaluated. Blood samples were collected from immunized mice two weeks after the second immunization and virus-specific antibody titers were measured by use of an ELISA. The black lines represent the mean of the antibody titers (n=3).

**Figure S2. Body weight changes and survival rates of immunized mice after lethal challenge (33 hit compounds).** Six-week-old BALB/c mice were immunized with PBS, compound alone, HA vaccine alone, or compound-adjuvanted HA vaccine twice with a two-week interval between vaccinations. Mice were intranasally challenged with 10 MLD_50_ of MA-CA04 virus three weeks after the second immunization. Body weight changes and survival rates were monitored daily for 14 days. The body weight data shown are means and standard deviations (SD) (n=4). Green asterisks indicate a significant difference between the vaccine alone and the vaccine plus compound group (vaccine plus compound *versus* vaccine alone); purple asterisks indicate a significant difference between the vaccine plus alum and the vaccine plus compound group (vaccine plus alum *versus* vaccine plus compound); * *P* <0.05.

**Figure. S3. Virus-specific antibody titers in sera of individual mice immunized with HA vaccine plus alum in the primary and secondary screens.** Six-week-old BALB/c mice were immunized with alum-adjuvanted HA vaccine twice with a two-week interval between vaccinations. Blood samples were collected from immunized mice two weeks after the second immunization and virus-specific antibody titers were measured by use of an ELISA. (A) Antibody titers of individual mice in the HA vaccine plus alum (positive control) groups for the 15 sets in the primary screen, the black lines represent the mean of the antibody titers (n=3 or 4); (B) Antibody titers of individual mice in the HA vaccine plus alum (positive control) groups for the 10 sets in the secondary screen, the black lines represent the mean of the antibody titers (n=4).
