# Supplementary material for: Food Additives as Novel Influenza Vaccine Adjuvants
Source: Vaccines (Basel). 2019 Sep 24;7(4):127. doi: 10.3390/vaccines7040127 (PMC6963695; doi:10.3390/vaccines7040127)
Supplement: Supplementary file 1 [file vaccines-07-00127-s001.zip › Supplementary Files/Feng_Vaccines_rev3_Sup Figs.pptx]

## Slide 1
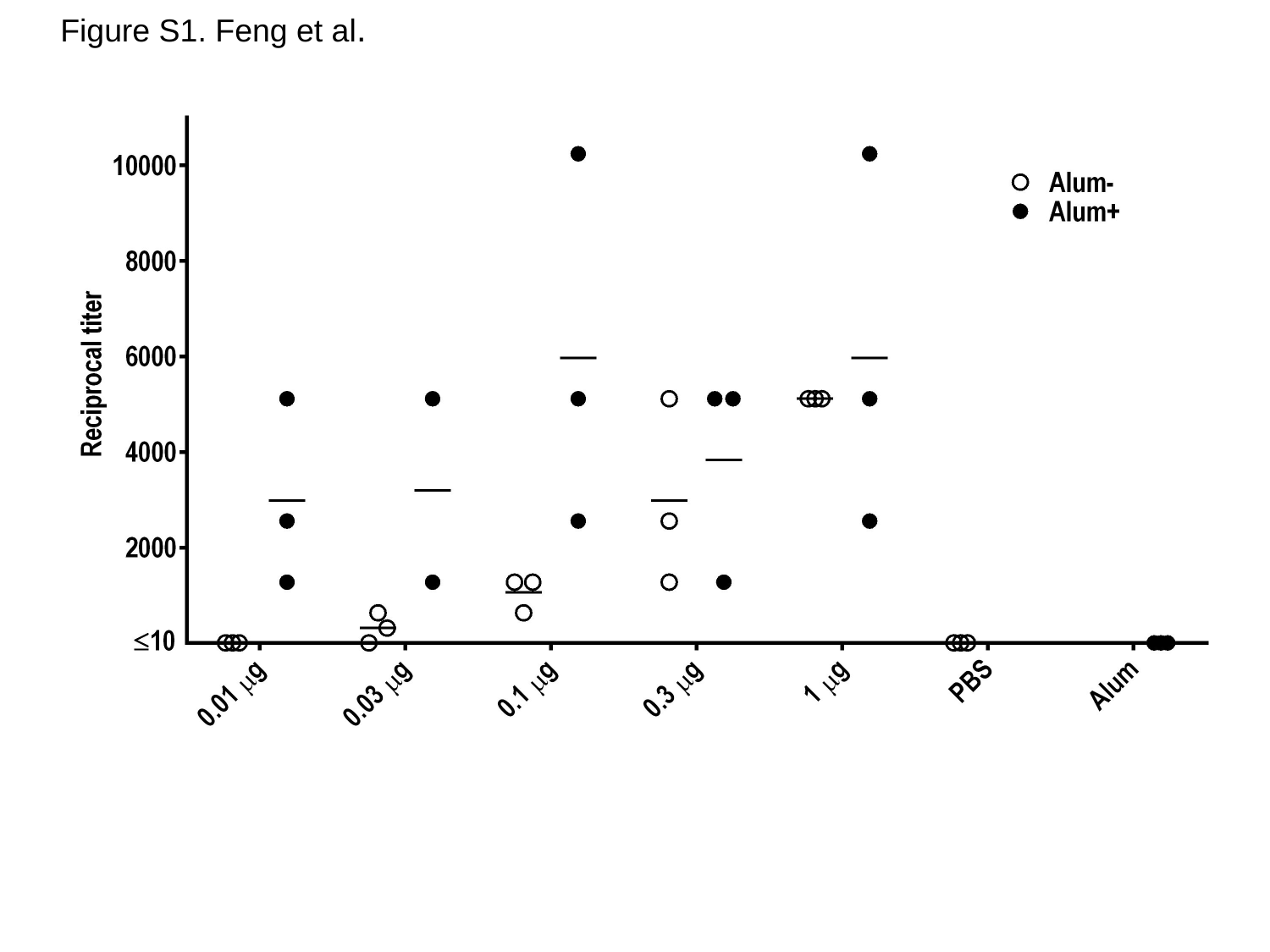

Figure S1. Feng et al.

## Slide 2
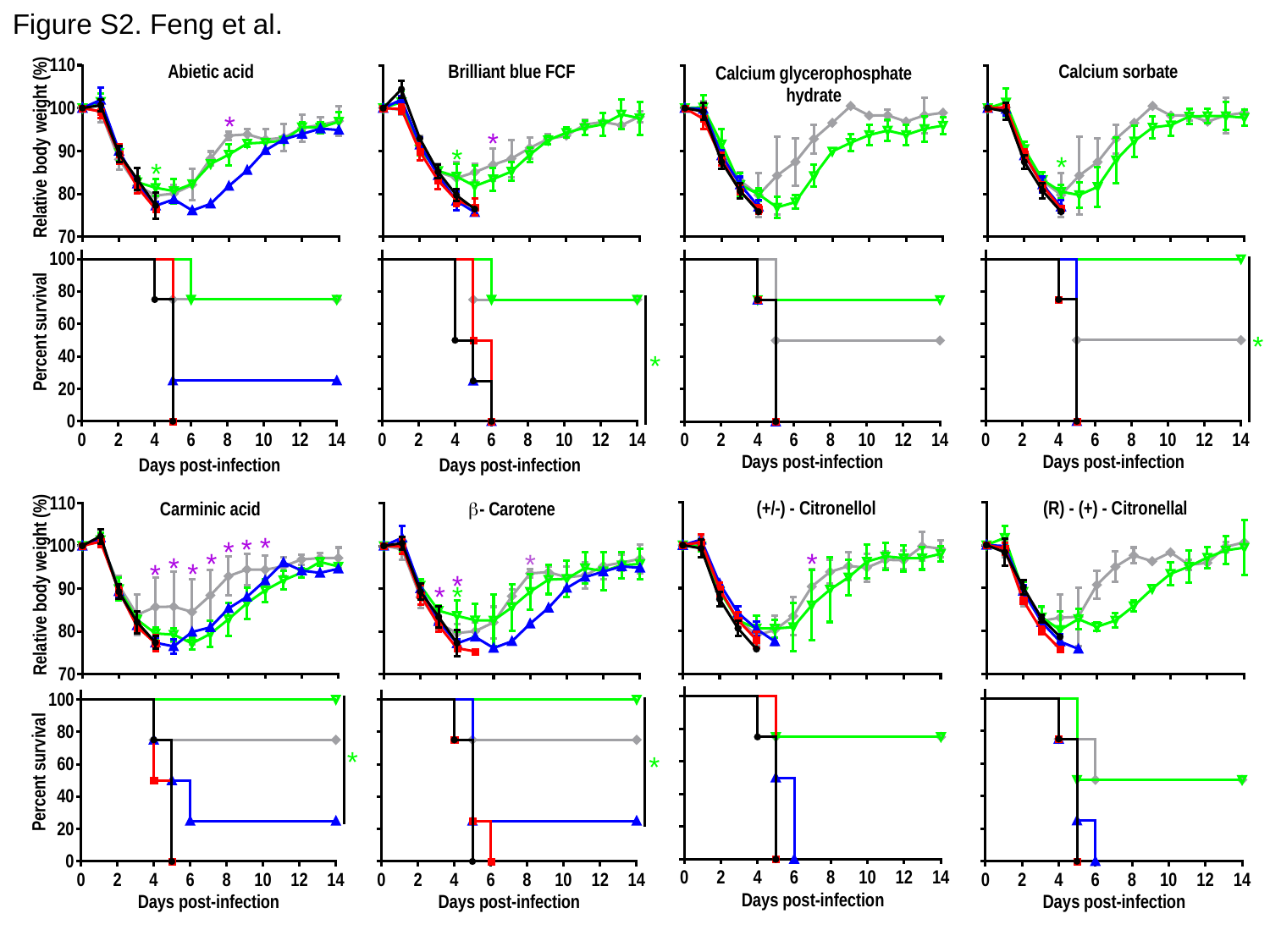

Figure S2. Feng et al.

## Slide 3
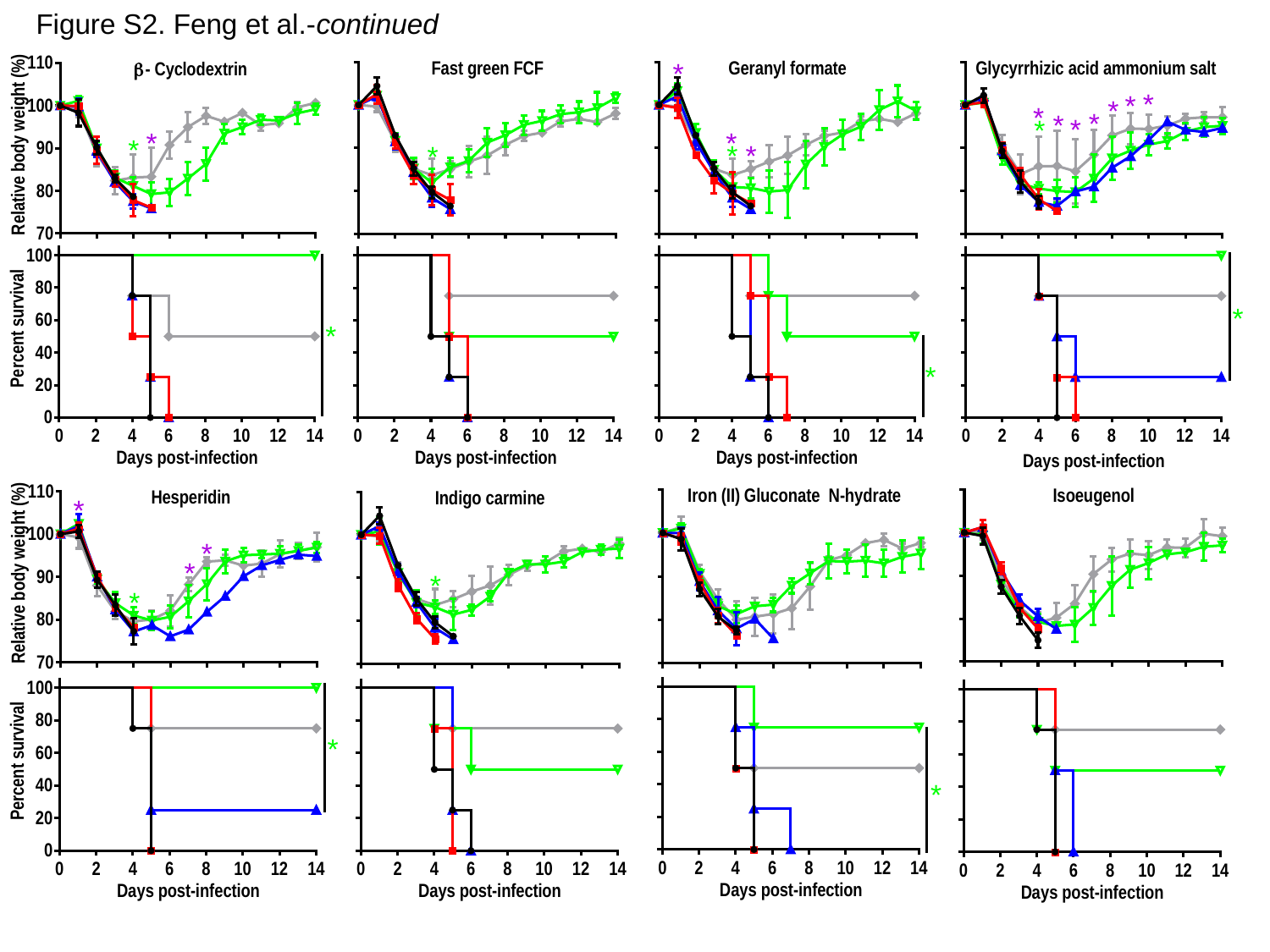

Figure S2. Feng et al.-continued

## Slide 4
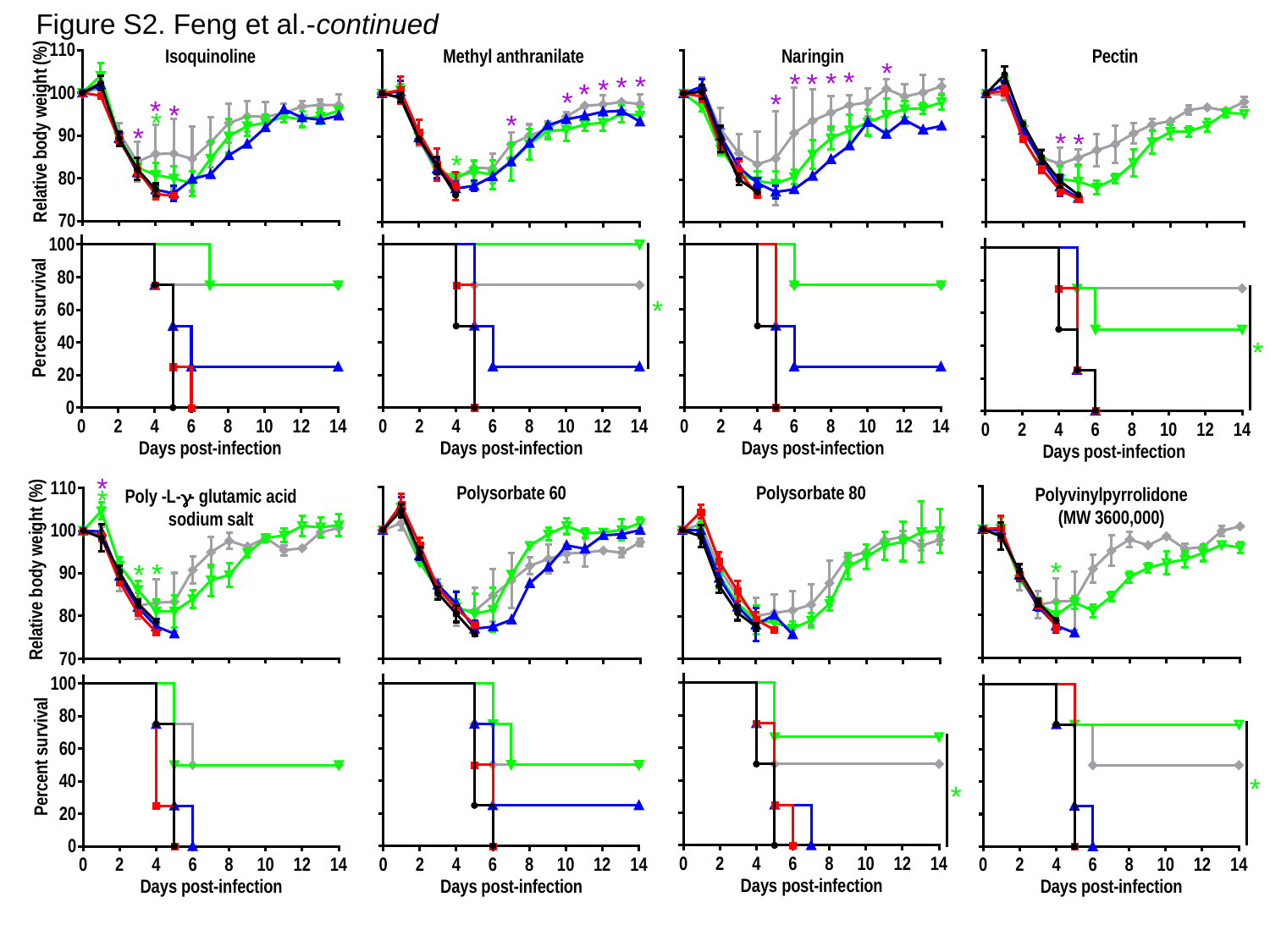

Figure S2. Feng et al.-continued

## Slide 5
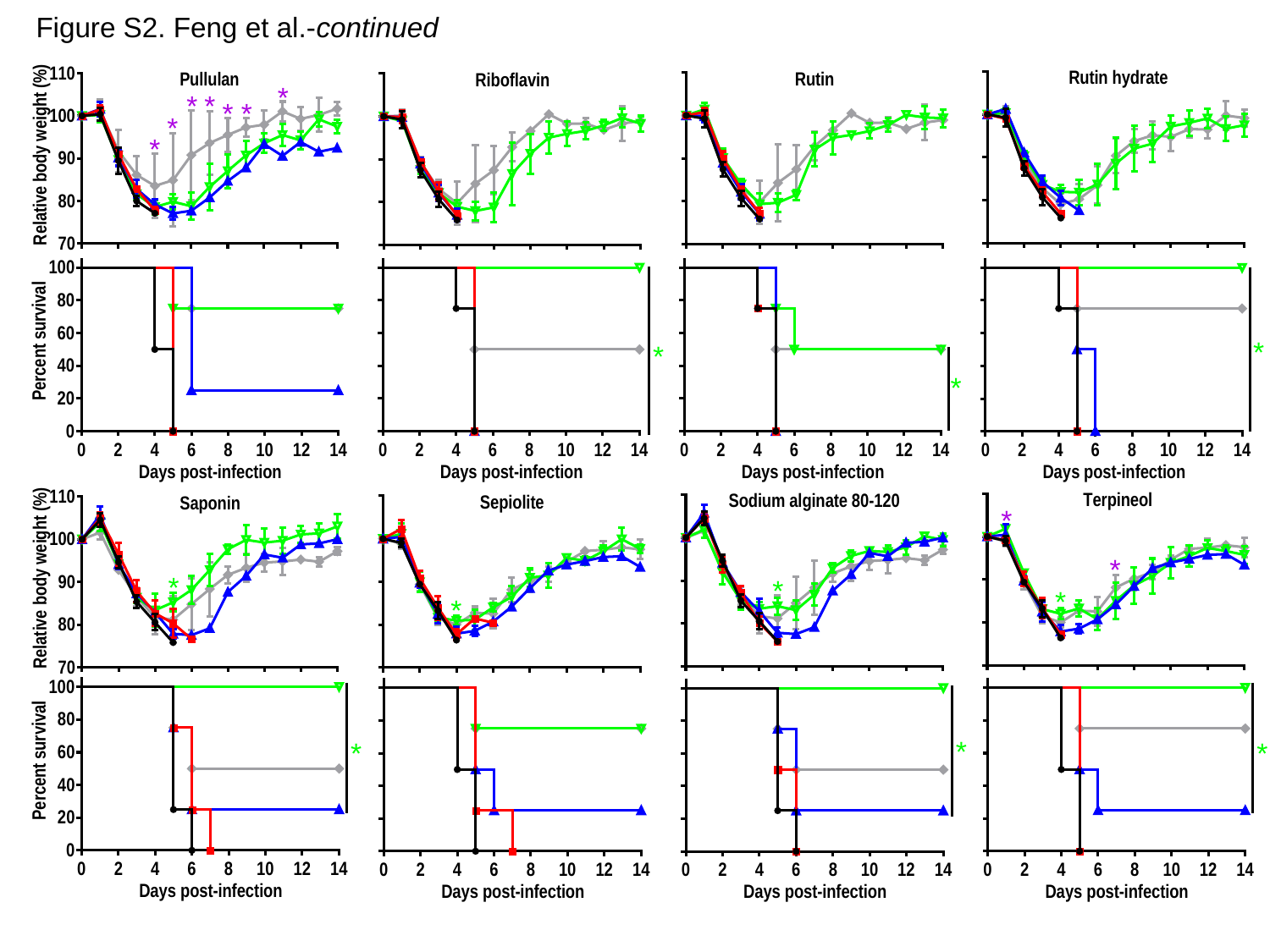

Figure S2. Feng et al.-continued

## Slide 6
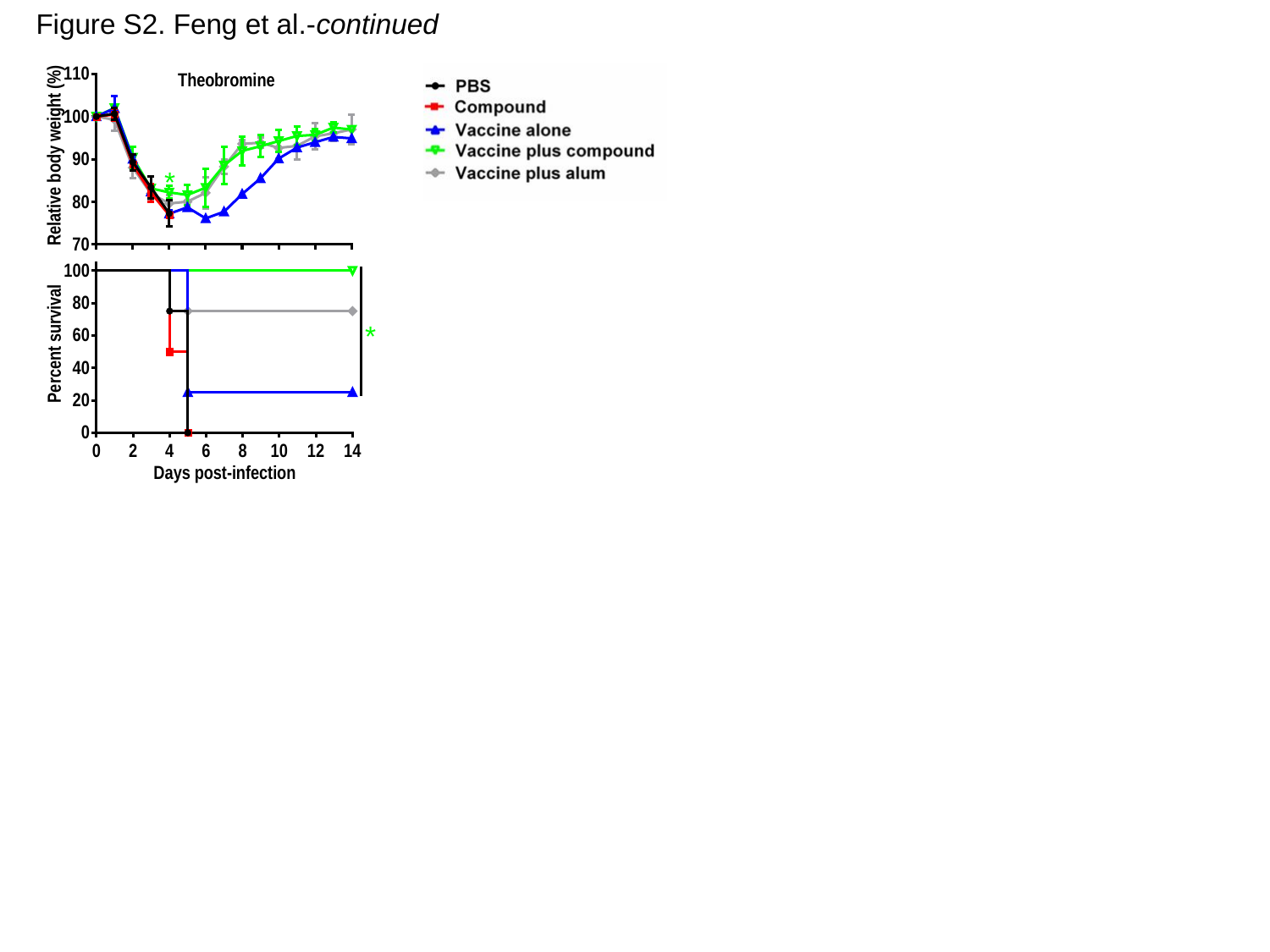

Figure S2. Feng et al.-continued

## Slide 7
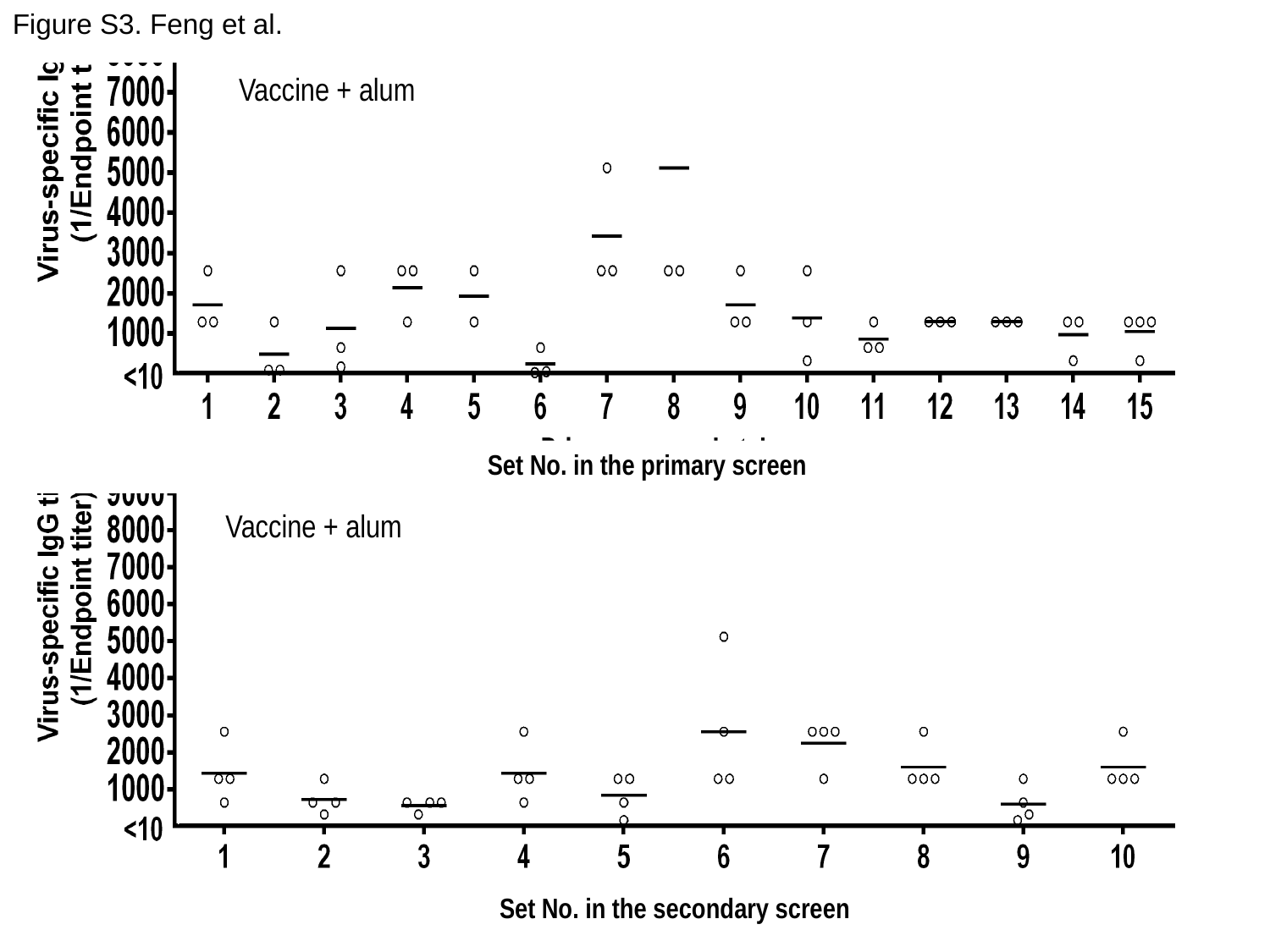

Figure S3. Feng et al.
Vaccine + alum
Set No. in the primary screen
Vaccine + alum
Set No. in the secondary screen
